# Coinfection of HIV and hepatitis C increases complication rates after total joint arthroplasty

**DOI:** 10.1051/sicotj/2020035

**Published:** 2020-09-19

**Authors:** Christopher Fang, Ella Cornell, Quinten Dicken, David Freccero, David Mattingly, Eric L. Smith

**Affiliations:** 1 New England Baptist Hospital 125 Parker Hill Ave Boston 02120 MA USA; 2 Boston Medical Center One Boston Medical Center Pl Boston 02118 MA USA

**Keywords:** HIV/AIDS, HCV, Coinfection, Total hip arthroplasty, Total joint arthroplasty, Total knee arthroplasty

## Abstract

*Introduction*: As advances in efficacy of human immunodeficiency virus (HIV) and hepatitis-C virus (HCV) anti-viral medications increase, patients are able to maintain higher quality of lives than ever before. While these patients live longer lives, the unique patient population of those co-infected with both HIV and HCV increases. As these older patients seek orthopaedic care, it is important to understand their unique outcome profile. The purpose of this study was to evaluate the complication rate after total joint arthroplasty (TJA) in patients with HIV and HCV coinfection compared with patients with HIV or HCV only. *Methods*: A retrospective review of patients undergoing primary total joint arthroplasty (TJA) at our urban, academic hospital between April 2016 and April 2019 was conducted. Patients were stratified into three groups according to viral status: HIV only, HCV only, or HIV and HCV coinfection. Baseline demographics, intravenous drug (IV) use, surgery type, CD4+ count, follow-up and complications were analysed. *Results*: Of the 133 patients included in the study, 28 had HIV, 88 had HCV and 17 were coinfected with both HIV and HCV. Coinfected patients were more likely to have a lower BMI (*p* < 0.039) and a history of IV drug use (*p* < 0.018) compared to patients with either HIV or HCV only. Coinfected patients had a higher complication rate (41%) than both HIV only (7%; *p* < 0.001) and HCV only (12.5%; *p* < 0.001) patients. *Discussion*: Patients coinfected with HIV and HCV undergoing TJA have a higher complication rate than patients with either infection alone. As this unique population of coinfected patients continues to expand, increasingly they will be under the care of arthroplasty surgeons. Improved awareness and understanding of the baseline demographic differences between these patients is paramount. Recognition of the increased complication rates grants the opportunity to improve their orthopaedic care through preoperative and multidisciplinary management.

## Introduction

The Center for Disease Control estimates over 1,000,000 individuals in the United States are infected with human immunodeficiency virus (HIV), and numbers are expected to continue to trend upwards [1]. Approximately 1.1% of people worldwide are infected with hepatitis C virus (HCV), higher than the 35 million individuals infected with HIV globally [2, 3]. Both patient populations have benefited from anti-viral medication advances, and thus are leading longer and higher quality lives [4, 5]. As a result, it is expected that more of these patients will be seeking total joint arthroplasty (TJA) care. Several studies have reported on the outcomes of these separate patient groups, showing increased risks of postoperative complications [6–11]. Furthermore, there exists a significant overlap in patients between these two populations, namely, patients with both HIV and HCV coinfection. Estimates suggest one out of four patients with HIV is also infected with HCV [3, 12–15]. Moreover, it is reported that the odds of a patient with HIV acquiring HCV increased 4% every year between 2002 and 2013 [16]. This coinfection rate increases to 90% when a patient has a history of intravenous (IV) drug use [13, 17, 18]. Therefore, it is important to understand the complication risks of this unique patient subset regarding TJA compared to the already increased odds of mono-infection. The aim of this study was to evaluate complication rates for HIV and HCV coinfected patients contrasted to patients with either HIV or HCV only. Our hypothesis is that patients with coinfection will have an increased rate of complications.

## Material and methods

### Study design

After Institutional Review Board (IRB) approval was obtained, a retrospective review of all patients diagnosed with HIV and/or HCV who underwent primary, unilateral total hip arthroplasty (THA) or total knee arthroplasty (TKA) between April 2016 and April 2019 at our urban, academic hospital was performed. Complications and follow-up time, in addition to demographic information regarding patient age, sex, body mass index (BMI), race/ethnicity, surgery, history of IV drug use and CD4+ count were collected. Patients were grouped into HIV only, HCV only and HIV + HCV coinfection according to viral diagnosis.

### Statistical analysis

Fisher’s exact tests and Student’s *t*-tests were appropriately used to compare categorical and continuous data between groups. All statistical analyses were performed using SAS v9.4 (SAS Institute, Cary, NC). Significance was defined as *p* < 0.05.

## Results

The study included 133 patients, with 28 patients in the HIV only group, 88 patients in the HCV only group and 17 patients in the HIV + HCV coinfection group. Patients across all groups did not differ in age, sex, surgery type, CD4+ count or follow-up (Table 1). The HIV + HCV coinfection group had a significantly lower BMI average than the HIV only (*p* = 0.039) and HCV only (*p* = 0.001) groups (Table 1). The HIV only group had significantly more Black/African Americans than the HCV only group (*p* < 0.001). The HCV only group had a higher rate of IV drug use than the HIV only group (*p* = 0.004), but the HIV + HCV coinfection group had a higher rate of IV drug use than both the HIV (*p* < 0.001) and HCV (*p* = 0.018) only groups (Table 1).

**Table 1:** Demographic information. Both = HIV + HCV coinfection.

	HIV (*n* = 28)	HCV (*n* = 88)	HIV + HCV (*n* = 17)	HIV × HCV	HIV × Both	HCV × Both
Age	56	58	61	*p* = 0.34	*p* = 0.087	*p* = 0.21
Female sex (%)	39.3%	37.5%	41.6%	*p* = 1.00	*p* = 0.54	*p* = 0.59
Body mass index (kg/m^2^)	29.30	31.03	25.88	*p* = 0.16	*p* = 0.039*	*p* = 0.001*
Race Black/African American	71.4%	31.8%	58.8%	*p* < 0.001*	*p* = 0.52	*p* = 0.052
IV drug use	14.3%	44.3%	76.5%	*p* = 0.004*	*p* < 0.001*	*p* = 0.018*
TKA’s	50%	46.6%	29.4%	*p* = 0.83	*p* = 0.22	*p* = 0.29
CD4+ count (cells/mm^3^)	562 (222–1022)	n/a	514 (16–1168)	n/a	*p* = 0.60	n/a
Follow up days	285.57	370.66	339.59	*p* = 0.08	*p* = 0.40	*p* = 0.29

* Denotes significance.

The HIV only group complication rate was 7% (*n* = 2), HCV only group complication rate was 12.5% (*n* = 10), and HIV + HCV coinfection group complication rate was 41% (*n* = 7) (Table 2). Complication rate of the HIV + HCV coinfection group was significantly higher than the HIV (*p* < 0.001) and HCV only (*p* < 0.001) groups (Table 2). Complications for HIV only group was surgical site infections (SSIs), HCV only group were SSIs, deep infections, aseptic loosening and instability and HIV + HCV coinfection group were SSIs, aseptic loosening, nerve palsy and dislocation.

**Table 2:** Complication rates for HIV only, HCV only and HIV + HCV. Both = HIV + HCV coinfection.

	HIV (*n* = 28)	HCV (*n* = 88)	HIV + HCV (*n* = 17)	HIV × HCV	HIV × Both	HCV × Both
Complication rate	7%	12.5%	41%	*p* = 0.73	*p* < 0.001*	*p* < 0.001*

* Denotes significance.

## Discussion

Patients coinfected with both HIV and HCV had higher complication rates than monoinfected patients with either HIV or HCV undergoing TJA at our institution. Coinfected patients had a significantly lower BMI and higher rates of IV drug use than either monoinfected patient cohort. These results are consistent with the literature looking at differences between coinfected and monoinfected patients, with coinfection being associated with lower BMI and increasing years of IV drug use [13, 17, 18]. To our knowledge, this is the first study including BMI into the analysis of coinfected patients compared to monoinfected patients undergoing TJA. The CD4+ count of coinfected and HIV only patients were similar, which could be attributed to all patients with an HIV diagnosis being treated with HAART at our institution.

Patients with HCV or HIV infection have an increased risk of postoperative complications following TJA [19, 20]. In a large retrospective database study, Kildow et al. demonstrated both HIV and HCV to be associated with increased postoperative complications, with HCV conferring a higher risk. Our results appear to reflect these data, with our HCV cohort having a 5.5% higher complication rate. It has also been shown that a majority of TJA HIV patients are younger on average and undergo THA (up to 79%) [11, 21]. Our data do not reflect this surgical distribution, with half of HIV patients undergoing THA, but do reflect the age distribution with our HIV cohort averaging 56 years at age of surgery, much lower than the usual age of 68 years for THA [11, 22].

This unique patient population of HIV and HCV coinfection is an important topic to approach. These patients are often at an increased risk of hip fractures due to often having reduced bone mineral density [23]. In addition, coinfected patients are generally younger, male, homeless and have higher rates of substance abuse, IV drug use, psychiatric disorders and readmission after surgery compared to uninfected patients [23–25]. Our findings were consistent with our cohort of coinfected patients similarly younger, more often male and with higher rates of IV drug use when compared to their controls [24, 25]. Further, coinfected patients have previously been shown to have an increased risk of complications after THA and longer hospital stays after surgery compared to patients without HIV or HCV infection [25]. Our coinfected cohort demonstrated an increased complication rate after TJA compared to their controls as well, further supporting these results. This current study represents an important example of evidence for the unique coinfected patient cohort. Many studies have demonstrated that patients infected with either HIV or HCV are at increased risk of complications after TJA [6–11], but studies regarding coinfected patients to draw conclusive management strategies are limited.

Two such studies that have previously looked at coinfection evaluated outcomes for patients with coinfection after TKA and THA using a large database [24, 25]. For coinfected patients undergoing TJA, they found increased length of hospital stay, hospital charges, multiple in-hospital complications, 90-day hospital readmissions and postoperative complications when compared to monoinfected and noninfected patient cohorts [24, 25]. Further supporting these findings, our study found similar results of coinfected patients displaying higher rates of postoperative complications compared to monoinfected patients. These data illustrate that not only do coinfected patients have an elevated risk of complications after TJA when compared to noninfected patients but also to monoinfected HIV or HCV patients as well. This warrants a heightened sense of appreciation for the risk profile of these coinfected patients, and future studies should evaluate optimal management strategies for this rare subset of patients.

Our current study has several limitations. First, we did not to include a control group to contrast our patient groups. Despite this, we were able to show significant differences of coinfected patients from monoinfected HIV and HCV patient cohorts, for which there is a clear pattern of increased complication risks when compared to controls in the literature [6–11, 24, 25]. Second, our mean duration of follow-up was less than a year (335 days), with minimal follow-up of 30 days. This was possibly due to socioeconomic barriers to follow-up appointments these patients faced. As these cases are limited, we chose to include these short-term results in our analysis. Lastly, our patient cohorts represented a limited sample size, especially for the coinfection group (*n* = 17), but this may mirror the true incidence of these rare patients. This infrequent distribution is further illustrated by a study of over 130,000 TJA subjects over four years, with only 68 patients (0.05%) in their coinfection group [24].

Our current study had a number of strengths. It evaluates the association between complication rate and HIV, HCV and coinfected patients at an urban academic hospital with a high volume of arthroplasty procedures. Another highlight includes a single institution study with a uniquely high patient population of interest compared to other proportions [24, 25] in a relatively short study period of 3 years. This helps to standardize the procedure protocol and outcomes to eliminate confounding variables that may arise from multi-centre variations. In addition, granular data on BMI were included in our analysis, which were not available for large database studies [23–25]. We believe this is the first evidence for an association between coinfected patients and a decreased BMI compared to monoinfected patients for those undergoing TJA.

### Conclusions

In our study, we found patients coinfected with HIV and HCV undergoing TJA have a higher complication rate than patients with either HIV or HCV alone. This expanding and vulnerable patient population will continue to seek TJA care and thus, it is essential to appreciate the distinct risk profile. To modify risks, multimodal care should be utilized with a focus on increasing BMI and ceasing IV drug use. This understanding will allow optimal care through management strategies such as incorporating interdisciplinary attention and higher priority preventative measures for the care of these patients undergoing TJA.

## Conflict of interest

The authors declare that they have no conflict of interest of any kind in connection with this article.
